# Acute oral dose of sodium nitrite induces redox imbalance, DNA damage, metabolic and histological changes in rat intestine

**DOI:** 10.1371/journal.pone.0175196

**Published:** 2017-04-06

**Authors:** Fariheen Aisha Ansari, Shaikh Nisar Ali, Hussain Arif, Aijaz Ahmed Khan, Riaz Mahmood

**Affiliations:** 1 Department of Biochemistry, Faculty of Life Sciences, Aligarh Muslim University, Aligarh, Uttar Pradesh, India; 2 Department of Anatomy, Faculty of Medicine, Jawaharlal Nehru Medical College, Aligarh Muslim University, Aligarh, Uttar Pradesh, India; Universita degli Studi di Napoli Federico II, ITALY

## Abstract

Industrialization and unchecked use of nitrate/nitrite salts for various purposes has increased human exposure to high levels of sodium nitrite (NaNO_2_) which can act as a pro-oxidant and pro-carcinogen. Oral exposure makes the gastrointestinal tract particularly susceptible to nitrite toxicity. In this work, the effect of administration of a single acute oral dose of NaNO_2_ on rat intestine was studied. Animals were randomly divided into four groups and given single doses of 20, 40, 60 and 75 mg NaNO_2_/kg body weight. Untreated animals served as the control group. An NaNO_2_ dose-dependent decline in the activities of brush border membrane enzymes, increase in lipid peroxidation, protein oxidation, hydrogen peroxide levels and decreased thiol content was observed in all treated groups. The activities of various metabolic and antioxidant defense enzymes were also altered. NaNO_2_ induced a dose-dependent increase in DNA damage and DNA-protein crosslinking. Histopathological studies showed marked morphological damage in intestinal cells. The intestinal damage might be due to nitrite-induced oxidative stress, direct action of nitrite anion or chemical modification by reaction intermediates.

## Introduction

Nitrite was once considered to be inert, but is now recognized to play varied vital functions in different tissues of the human body, at low physiological concentration. It is also a ready source of nitric oxide (NO) which plays key role in immunology, physiology and neuroscience [1]. NO improves gastrointestinal health by mediating gastric blood flow; maintaining integrity of gastric epithelium and the mucus barrier and inhibiting leukocyte adherence to the endothelium [2]. The vasodilatory and hypotensive effect of NO is also known to decrease the risk of cardiovascular diseases and improve pulmonary health [1,3]. Sodium nitrite (NaNO_2_) is widely used in the food industry as color fixative and preservative of fish and meat products. It acts as a flavour-enhancer and retards rancidity by preventing fat oxidation. It also inhibits the growth of micro-organisms, especially *Clostridium botulinum* which causes botulism. NaNO_2_ is used for dye synthesis, manufacture of rubber chemicals and nitroso compounds and has several other industrial purposes. Medicinally, it is used for vasodilation, bronchodilation and as antidote for cyanide poisoning.

Several potential health benefits of nitrite have been reported at low physiological concentrations (0.45–23 μM) [4]. However, at high concentrations, or chronic exposure to even low doses, nitrite is known to be detrimental to health, even causing death in some cases. Anthropogenic activities have greatly increased the nitrate-nitrite content of the environment. Rampant use of nitrogenous fertilizers to increase crop yield and improper treatment of industrial and sewage wastes are the primary contributing factors [5]. Many areas across the globe have reported nitrate/nitrite content in drinking water that greatly exceeds the acceptable limits of 1 ppm (nitrite) and 10 ppm (nitrate) set by the U.S. Environmental Protection Agency [6,7].

Accidental or intentional acute exposure to high levels of nitrite has been reported to cause death, mainly due to methemoglobinemia [8,9]. Chronic exposure to lower doses of nitrite causes adverse health effects, which includes birth defects, respiratory tract ailments, damage to the nervous system and paralysis [10]. Prolonged exposure to nitrite can also cause carcinogenicity and mutagenicity [11,12]. The fraction of population which is more prone to nitrite toxicity includes anemic and glucose 6-phosphate dehydrogenase (G6PD) deficient individuals, pregnant women and infants [6].

Human exposure to nitrite mainly occurs through the oral route. Nitrite taken through contaminated drinking water or food, primarily affects the gastrointestinal tract and small intestine. Quantitatively, absorption through the gut is greater than other routes (bioavailability >92%) as nitrite is rapidly and almost completely absorbed through the small intestine [13]. The acidic pH (<2) environment of the gut greatly favors the conversion of nitrite into a nitrosating agent, which may result in the formation of nitrosamines. Nrisinha et al. [14] have reported that cured meat forms N-nitroso-N-methylurea following incubation with nitrite at gastric pH. NO, from nitrite, generates peroxynitrite and nitryl chloride upon reaction with superoxide radical and hypochlorous acid, respectively. These species are even more damaging than nitrite and can act as direct mutagens [15]. NaNO_2_ treatment increases micronucleated cells and chromatid gaps in lymphocytes and causes mutagenicity in *Salmonella typhimurium* strain TA100 [16].

Oxidative damage is considered to be one of the main mechanism by which nitrite exerts its toxicity. Several *in vivo* and *in vitro* studies have reported nitrite toxicity mediated through oxidative stress [17,18]. This is supported by reports that antioxidants can ameliorate nitrite toxicity [19,20]. Ozen et al. [21] have reported degenerative changes in organs of nitrite-treated mice. Although chronic exposure of humans to low doses of nitrite is common world-wide, many cases of intentional or accidental exposure to high doses of nitrite have also been documented [22,23]. However, reports on the mechanistic details of nitrite-induced oxidative stress in mammals are lacking in literature. In light of this, the present work was undertaken to analyse the effect of a single acute oral dose of NaNO_2_ on DNA, morphological and biochemical aspects of rat intestine.

## Materials and methods

### Chemicals

Agarose, 1-chloro-2,4-dinitrobenzene, 2,2-diphenyl-1-picrylhydrazyl (DPPH), N-ethylmaleimide, glutathione reductase (GR), L-leucine *p*-nitroanilide, metaphosphoric acid, NaNO_2_, Roswell Park Memorial Institute 1640 (RPMI) medium, Triton X-100, 2,4,6-tris(2-pyridyl)-*s*-triazine, p-nitrophenyl phosphate, γ-glutamyl p-nitroanilide, o-phthalaldehyde and NaNO_2_ were from Sigma Aldrich, USA. Bovine serum albumin, diphenylamine, 5,5'-dithiobisnitrobenzoic acid (DTNB), ethylenediaminetetraacetic acid (EDTA), glucose 6-phosphate, proteinase K, reduced (GSH) and oxidized (GSSG) glutathione, hydrogen peroxide (H_2_O_2_), reduced and oxidized nicotinamide adenine dinucleotide phosphate (NADPH and NADP^+^), sodium dodecyl sulfate (SDS), sorbitol, tris(hydroxymethyl)aminomethane (Tris), pyrogallol, 2,4-dinitrophenylhydrazine and xylenol orange were from Sisco Research Laboratories, India. Thiobarbituric acid and trichloroacetic acid were from Himedia Laboratories (Mumbai, India).

All other chemicals used were of analytical grade.

### Experimental protocol

Animal housing and handling was conducted as per the institutional guidelines (Registration number: 714/GO/Re/S/02/CPCSEA) and all experimental procedures were approved by the Institutional Ethics Committee (IEC) of Aligarh Muslim University that monitors research involving animals. All efforts were made to minimize any suffering of rats during the entire treatment period. Adult male Wistar rats weighing 160–180 gm were used in this study. Animals were acclimatized for seven days on standard rat pellet diet and water *ad libitum* and then divided into five groups of six animals each (one control and four NaNO_2_ treated groups). Animals in each treated group received a single acute dose of NaNO_2_ (given orally through gavage) at 20, 40, 60 and 75 mg/kg body weight. Animals in the control (untreated group) were given an equivalent volume of water through gavaging. No animal died during the treatment period since the doses were below the reported LD_50_ value of NaNO_2_ (85–150 mg/kg body weight) for rats. All animals had free access to water and food during the entire treatment period and were sacrificed 24 h after administration of NaNO_2_, under local anesthesia using diethyl ether. The entire small intestine was removed, cleaned thoroughly by flushing with ice-cold 0.9% NaCl and then slit open along its entire length. A glass slide was used to gently scrape the mucosal layer and the scrapings used for the preparation of homogenates and brush border membrane (BBM) vesicles. For DNA damage studies, small intact portions of the intestine were separately suspended in RPMI (for comet assay) or homogenized in suitable buffer.

### Preparation of mucosal homogenates and BBM vesicles

A 10% (w/v) homogenate of the mucosal scraping was prepared in 2 mM Tris–HCl, 50 mM mannitol buffer, pH 7.0, using an Ultra Turrex Kunkel homogenizer and glass Teflon homogenizer, by passing five pulses of 30 s each. Aliquots were immediately stored at -20°C for the analysis of non-enzymatic parameters. The homogenates were centrifuged separately at 400 × g and 2,400 × g at 4°C for 15 min and the supernatants collected and stored at -20°C for the analysis of various enzymatic activities. The CaCl_2_ precipitation method was used to prepare BBM vesicles from the homogenates, exactly as described by Farooq et al. [24]. The protein concentration in homogenates and BBM vesicles was determined by the method of Lowry et al. [25].

### Histopathology

Approximately 3 cm long duodenal section of the intestine was cut and fixed immediately in Karnovsky fixative. Thin microscopic sections of 5 μm were cut using a paraffin block. The cells were stained with hematoxylin and eosin and examined under a microscope (Olympus BX40, Japan) at 100x magnification [26].

### BBM enzymes

L-leucine p-nitroanilide, p-nitrophenyl phosphate and γ-glutamyl p-nitroanilide were used as substrates to determine the activities of leucine aminopeptidase (LAP), alkaline phosphatase (ALP) and γ-glutamyl transferase (GGT), respectively [27–29]. Sucrase activity was determined by the reaction between reducing sugars, produced upon hydrolysis of sucrose by the enzyme, and 3,5-dinitrosalicylic acid to form a colored product [30].

Kinetic studies were done using isolated BBM vesicles. The enzymes were assayed at different substrate concentrations which are given: LAP: 0.3–2 mM L-leucine p-nitroanilide; ALP: 4–25 mM p-nitrophenyl phosphate; GGT: 0.33–2 mM γ-glutamyl *p*-nitroanilide; sucrase: 5–32 mM sucrose. The data was analyzed by double reciprocal (1/v vs 1/[S]) Lineweaver-Burk plots, K_M_ (Michaelis constant) and V_max_ (maximum velocity) values were obtained for each enzyme.

### Lipid peroxidation, protein oxidation, GSH, total SH content and H_2_O_2_ levels

Several non-enzymatic parameters of oxidative stress were determined in crude intestinal homogenates. Lipid peroxidation was quantified from the reaction of its end product malondialdehyde with thiobarbituric acid [31] and the results are expressed as TBARS (thiobarbituric acid reactive substances). 2,4-Dinitrophenyl hydrazine was used to determine carbonyl group content which serves as an index of protein oxidation [32]. Reduced glutathione (GSH) levels were determined by fluorometric analysis, using N-ethylmaleimide and o-phthalaldehyde [33]. Total sulfhydryl (SH) content was determined using DTNB. The reaction between SH groups and DTNB gives yellow colored thionitrobenzoate, whose absorbance was determined at 412 nm [34]. Hydrogen peroxide (H_2_O_2_) levels were quantified using FOX reagent (ferrous ammonium sulphate-xylenol orange) in the presence of 0.1 M sorbitol [35].

### Acid phosphatase(ACP) and total adenosine triphosphatase (ATPase) activity

ACP and total ATPase activities were determined in crude intestinal homogenates. ACP activity was measured from the enzyme catalysed hydrolysis of p-nitrophenyl phosphate, at pH 4.5, to form p-nitrophenol [36]. Total ATPase activity was assayed by quantifying the inorganic phosphate released upon ATP hydrolysis [37].

### Antioxidant power

The antioxidant power was determined in intestinal homogenates by FRAP (ferric reducing/antioxidant power) and DPPH (2,2-diphenyl-1-picrylhydrazyl) assays. These assays measure ferric reducing and free radical quenching ability of the cells using electrons donated by antioxidants in the sample. In the FRAP assay, 100 μl intestinal homogenate was incubated with 1.5 ml of FRAP reagent (containing 2,4,6-tris(2-pyridyl)-s-triazine and FeCl_3_ in sodium acetate buffer, pH 3.6) at room temperature. After 5 min, the absorbance at 593 nm was noted and readings quantitated using a standard of ferrous sulphate [38]. DPPH assay was performed by mixing 0.4 ml of 10 mM sodium phosphate buffer, pH 7.4, with 0.1 ml intestinal homogenate followed by addition of 0.5 ml of 0.1 mM solution of DPPH in methanol. The reaction mixture was left in the dark for 30 min at 21°C. After centrifugation at 12,000 × g, absorbance of the supernatants was read at 517 nm [39]. A 0.05 mM solution of DPPH was taken as reference. The DPPH scavenging capacity was calculated as:

%QuenchingofDPPH=[1-(Absorbanceofsample/Absorbanceofreference)]x100

### Enzymes involved in antioxidant defense mechanism

The supernatants of intestinal homogenates collected after centrifugation at 2,400 × g for 15 min at 4°C were used to determine the activities of enzymes involved in free radical scavenging. Cu,Zn superoxide dismutase (SOD) was assayed from the inhibition of pyrogallol auto-oxidation by the enzyme [40] and catalase (CAT) from the decomposition of H_2_O_2_ to water [41]. Glutathione reductase (GR) catalyzes the reduction of oxidized glutathione to its reduced form with concomitant conversion of NADPH to its oxidized form (NADP^+^) [42]. The enzyme activity was determined by monitoring the decrease in absorbance at 340 nm. The enzyme Thioredoxin reductase (TR) was assayed from the formation of yellow colored thionitrobenzoate anion produced upon reduction of DTNB in the presence of NADPH [43]. Glutathione peroxidase activity was determined by the method of Flohé and Günzler [44] by following the decrease in absorbance at 340 nm upon conversion of NADPH to NADP^+^.

### Enzymes of carbohydrate metabolism

The supernatants of intestinal homogenates collected after centrifugation at 400 × g for 15 min at 4°C were used to determine the activities of enzymes involved in carbohydrate metabolism. The activities of lactate dehydrogenase (LDH), malate dehydrogenase (MDH), glucose 6-phosphatase (G6Pase), fructose 1,6-bisphosphatase (F6Pase) and malic enzyme (ME) were assayed as described by Khundmiri et al. [45]. Hexokinase (HX) activity was determined by the method of Crane and Sols [46] and G6PD was assayed using glucose 6-phosphate as substrate and monitoring the reduction of NADP^+^ to NADPH at 340 nm [47].

### DNA damage studies

#### Alkaline comet assay

The assay was performed as described by Singh et al. [48] with slight modifications. The suspension of intestinal mucosal cells in RPMI medium was mixed with 0.1 ml of 1% molten low-melting-agarose and immediately pipetted onto agarose pre-coated slides. A second layer of agarose was applied over the cell suspension layer and allowed to solidify properly. Cells were lysed by immersing the slides in lysing solution (2.5 M NaCl, 100 mM EDTA, 10 mM Tris-HCl, 1% Triton X-100, pH 10.0) for 3 hours at 4°C. To allow DNA unwinding, slides were incubated for 30 min in alkaline electrophoresis buffer (1 mM EDTA, 300 mM NaOH, pH 13.0). Electrophoresis was carried out at 0.7 V/cm and 300 mA for 30 min at 4°C. The slides were then neutralized with cold 0.4 M Tris-HCl, pH 7.5. The cells were stained with ethidium bromide and analysed under a CX41 fluorescent microscope equipped with the image analysis system, Komet 5.5, Kinetic Imaging, Liverpool, UK. The comets were scored at a magnification of 100x and images of 50 cells (25 from each replicate slide) per sample were scored. Tail-lengths were recorded.

#### Quantitative DNA fragmentation

DNA fragmentation was assayed by the colorimetric diphenylamine method of Burton [49]. Results are expressed as percentage of fragmented DNA to total DNA.

#### Isolation of DNA and electrophoresis

Whole genomic DNA was isolated from mucosal tissue as described by Evans et al. [50]. Intestinal cells were lysed using lysis buffer (10 mM Tris-HCl, 0.1 M EDTA, 0.5% sodium dodecyl sulfate, pH 8.0) and equal volumes of phenol: chloroform: isoamyl alcohol solution (25: 24: 1) was added. The samples were centrifuged at 10,000 x g at 4°C and the aqueous layer containing DNA transferred to sterile micro-centrifuge tubes. The DNA was precipitated with ethanol, washed and resuspended in TE buffer (100 mM Tris-HCl, 10 mM EDTA, pH 8.0). The DNA was first treated with DNase free pancreatic RNase (20 μg/ml final concentration) and then with proteinase K (0.1 mg/ml final concentration). Contaminating proteins were again removed by phenol: chloroform extraction and DNA precipitated with ethanol, washed with ice-cold 70% ethanol. The pellet of DNA was finally resuspended in TE buffer. The isolated DNA was electrophoresed on 0.7% agarose gels, stained with ethidium bromide and visualized under UV light.

#### DNA-protein crosslinking (DPC)

Intestinal mucosal tissue was homogenized in Tris buffer (20 mM Tris-HCl, 20 mM EDTA, 2% SDS, pH 7.5) and the DPCs were detected by the K^+^-SDS assay exactly as described by Zhitkovich and Costa [51].

### Statistical analysis

All experiments were done three times to document reproducibility. The results of one set of experiments are presented here and are expressed as mean ± standard error of mean. Analysis of variance (ANOVA) was used in combination with Post-Hoc test (Bonferroni comparison test) using Origin Pro 8 software (USA) to evaluate the data. For clarity, the results of treatment groups were compared to the control group. Statistical significance is indicated at a probability level of p <0.05.

## Results

### BBM enzymes

The specific activities of four BBM enzymes were determined in intestinal mucosal homogenates and isolated BBM vesicles. NaNO_2_ administration resulted in significant decrease in the activities of all enzymes in intestinal homogenates. An NaNO_2_ dose-dependent decrease was evident with the lowest activity present in the highest dose group of 75 mg/kg body weight. The percentage decrease in the activities of the enzymes, compared to the control group, were- LAP, 62.3; GGT, 45; ALP, 53.3 and sucrase, 40.2 (Table 1). A similar trend was observed in BBM vesicles, the extent of decrease being almost the same in all BBM enzymes. In BBM vesicles from the highest dose group, the observed percentage decrease was- LAP, 59.5; GGT, 48.2; ALP, 55.4 and sucrase, 42.5 when compared to the control group (Table 2).

**Table 1:** Effect of NaNO_2_ on the activities of BBM marker enzymes in rat intestinal homogenates.

	Control	Dose of NaNO_2_ (per kg body weight)			
		20 mg	40 mg	60 mg	75 mg
LAP	6.1 ± 0.31	5.7 ± 0.29 (6.6)	5.1 ± 0.26* (16.4)	3.1 ± 0.16* (49.2)	2.3 ± 0.12* (62.3)
GGT	8.4 ± 0.43	7.8 ± 0.40 (7.1)	6.7 ± 0.35* (20.2)	4.9 ± 0.26* (41.7)	4.6 ± 0.21* (45)
ALP	3.0 ± 0.16	2.7 ± 0.14* (10)	2.2 ± 0.12* (26.7)	1.8 ± 0.10* (40)	1.4 ± 0.08* (53.3)
Sucrase	9.1 ± 0.47	8.6 ± 0.44 (5.5)	7.4 ± 0.38* (18.7)	5.9 ± 0.30* (35.2)	5.44 ± 0.28* (40.2)

Specific activities of all enzymes are in μmoles/mg protein/h. Values in parenthesis represent percent decrease from control.

Results are mean ± standard error of six different preparations.

* Significantly different at p < 0.05 from control.

LAP, leucine aminopeptidase; GGT, gamma-glutamyl transferase; ALP, alkaline phosphatase.

**Table 2:** Effect of NaNO_2_ on the activities of BBM marker enzymes in isolated rat intestinal BBM vesicles.

	Control	Dose of NaNO_2_ (per kg body weight)			
		20 mg	40 mg	60 mg	75 mg
LAP	33.8 ± 1.7	29.2 ± 1.6* (13.6)	25.7 ± 1.3* (24)	18.4 ± 1.0* (45.6)	13.7 ± 0.7* (59.5)
GGT	41.3 ± 2.1	38.7 ± 2.0 (6.2)	33.2 ± 1.8* (19.6)	25.4 ± 1.3* (38.5)	21.4 ± 1.2* (48.2)
ALP	18.6 ± 1.0	17.1 ± 0.9 (8.1)	13.6 ± 0.7* (26.9)	10.9 ± 0.6* (41.4)	8.3 ± 0.4* (55.4)
Sucrase	44.2 ± 2.3	41.4 ± 2.2* (6.3)	35.7 ± 1.8* (19.2)	29.5 ± 1.5* (33.3)	25.4 ± 1.3* (42.5)

Specific activities of all enzymes are in μmoles/mg protein/h. Values in parenthesis represent percent decrease from control.

Results are mean ± standard error of six different BBM preparations.

* Significantly different at p < 0.05 from control.

LAP, leucine aminopeptidase; GGT, gamma-glutamyl transferase; ALP, alkaline phosphatase.

**Fig 1:**
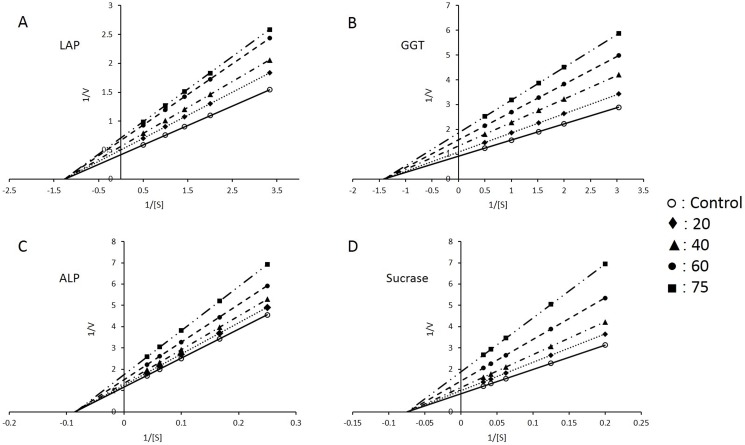
Kinetic studies with different enzymes in intestinal BBM vesicles. (A) Leucine aminopeptidase (LAP) (B) γ-glutamyl transferase (GGT) (C) alkaline phosphatase (ALP) and (D) sucrase. Enzyme activities were assayed at different substrate concentrations in samples from control, 20 mg, 40 mg, 60 mg and 75 mg/kg body weight NaNO_2_ treated groups. Data were analysed by double reciprocal 1/v vs 1/[S] Lineweaver-Burk plots. Results are mean ± standard error of six different BBM preparations.

Since there was significant inhibition (40–60%) of all BBM enzymes, kinetic studies were done by assaying them at different substrate concentrations and analysing the data by double reciprocal Lineweaver-Burk plots. These kinetic studies used isolated BBM vesicles and suggest non-competitive inhibition of all four enzymes by nitrite. For each enzyme, K_M_ (Michaelis constant) value remained same while V_max_ (maximum velocity) decreased in an NaNO_2_ dose-dependent manner (Fig 1).

**Fig 2:**
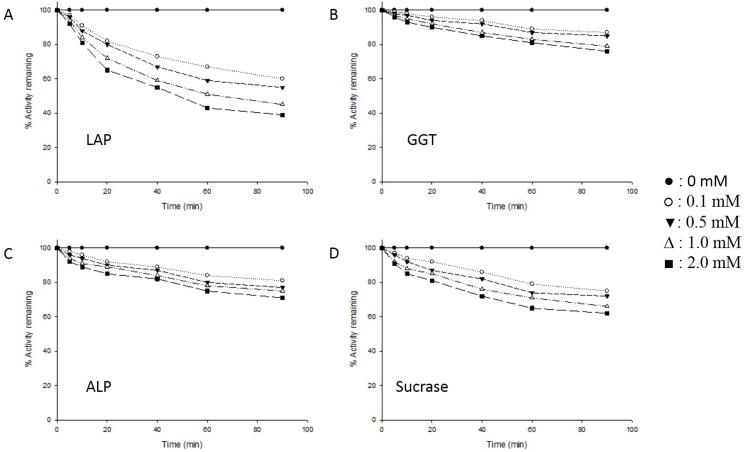
Effect of NaNO_2_ on the activities of enzymes of rat intestinal BBM vesicles, under *in vitro* condition. Intestinal BBM vesicles (1 mg protein/ml in 50 mM sodium maleate buffer, pH 6.0), from control animals were incubated with varying concentrations (0–2.0 mM) of NaNO_2_ at 37°C in a total reaction volume of 1.0 ml. At different time intervals after the addition of NaNO_2_, aliquots were removed from the reaction mixture and assayed for enzyme activity. Results are expressed relative to enzyme activity in untreated BBM vesicle samples kept on ice which served as the control. Results of a typical experiment are shown here. (A) Leucine aminopeptidase (LAP) (B) γ-glutamyl transferase (GGT) (C) alkaline phosphatase (ALP) (D) sucrase.

The probability of enzyme inhibition through direct interaction with nitrite was checked by performing an *in vitro* experiment. Isolated BBM vesicles from control animals were incubated with different concentrations of NaNO_2_ at 37°C and the activity of each enzyme determined at different time intervals. All four enzymes showed significant direct inhibition by nitrite. Under these conditions, LAP activity was the most affected, showing 61% inhibition, followed by sucrase (38% inhibition), ALP (29% inhibition) and GGT (24% inhibition). No loss of any enzyme activity was observed in untreated BBM vesicle samples incubated at 37°C, ruling out thermal inactivation of the enzymes (Fig 2).

### Lipid peroxidation, protein oxidation, GSH, total SH content and H_2_O_2_ levels

Increase in lipid peroxidation, protein oxidation and H_2_O_2_ levels was observed in NaNO_2_-administered rats, in a dose-dependent manner. GSH levels and total SH content were both lowered concomitantly indicating decreased reducing ability of the cell. In the highest NaNO_2_ dose group, the change in these parameters were- total SH content, -44.7%; GSH, -55%; lipid peroxidation, +163.6%; protein oxidation, +132.3% and H_2_O_2_, +117%, when compared to the control group (Table 3).

**Table 3:** Effect of NaNO_2_ on some non-enzymatic parameters of oxidative stress in rat intestinal homogenates.

	Control	Dose of NaNO_2_ (per kg body weight)			
		20 mg	40 mg	60 mg	75 mg
Total SH	0.47 ± 0.03	0.43 ± 0.02 (-8.5)	0.30 ± 0.02* (-36.2)	0.27 ± 0.02* (-42.6)	0.26 ± 0.02* (-44.7)
GSH	8.0 ± 0.40	7.1 ± 0.40* (-11.3)	6.3 ± 0.35* (-21.3)	4.1 ± 0.21* (-48.8)	3.6 ± 0.21* (-55)
LPO	0.11 ± 0.006	0.18 ± 0.01* (+63.6)	0.22 ± 0.01* (+100)	0.25 ± 0.01* (+127)	0.29 ± 0.02* (+163.6)
PO	3.1 ± 0.15	3.9 ± 0.20* (+25.8)	5.5 ± 0.30* (+77.4)	6.8 ± 0.35* (+119.4)	7.2 ± 0.38* (+132.3)
H_2_O_2_	0.24 ± 0.01	0.29 ± 0.02 (+21)	0.35 ± 0.02* (+45.8)	0.47 ± 0.03* (+95.8)	0.52 ± 0.03* (+117)

Total SH content is in μmoles/mg protein and GSH, LPO, PO and H_2_O_2_ are in nmoles/mg protein. Values in parenthesis represent percent change from control.

Results are mean ± standard error of six different preparations.

* Significantly different at p < 0.05 from control.

GSH, reduced glutathione; SH, sulfhydryl group; LPO, lipid peroxidation; PO, protein oxidation.

### ACP and total ATPase activity

NaNO_2_ differently altered the activities of ACP and total ATPase. The activity of ACP, a lysosomal marker enzyme, was found to decrease, while the activity of total ATPase, a membrane bound enzyme, was found to increase with increasing dose of NaNO_2_. The alterations in enzyme activities in intestinal homogenates of the highest dose group were- ACP, +157% and total ATPase, -56%, as compared to control group (Table 4).

**Table 4:** Effect of NaNO_2_ on acid phosphatase and total ATPase activities in rat intestinal homogenates.

	Control	Dose of NaNO_2_ (per kg body weight)			
		20 mg	40 mg	60 mg	75 mg
ACP	1.4 ± 0.07	1.8 ± 0.10 (+28.6)	2.5 ± 0.13* (+78.6)	3.2 ± 0.17* (+128.6)	3.6 ± 0.19* (+157)
Total ATPase	3.4 ± 0.18	3.0 ± 0.15* (-11.8)	2.3 ± 0.13* (-32.4)	1.9 ± 0.10* (-44.1)	1.5 ± 0.08* (-56)

Specific activities of both enzymes are in μmoles/mg protein/h. Values in parenthesis represent percent change from control.

Results are mean ± standard error of six different preparations.

* Significantly different at p < 0.05 from control.

ACP, acid phosphatase; ATPase, adenosine triphosphatase.

### Antioxidant power

Antioxidants in sample can serve as donors of electron which can quench free radicals or reduce metal ions to lower oxidation states. This was determined by employing the widely used FRAP and DPPH assays which serve as indicators of antioxidant power of cell. Both assays showed that the antioxidant capacity of NaNO_2_-administered animals decreased dose-dependently as compared to control animals. FRAP values were lowered by 54.2% and DPPH by 31.6% in the highest dose group, as compared to control values (Table 5).

**Table 5:** Effect of NaNO_2_ on antioxidant power of rat intestinal homogenates.

	Control	Dose of NaNO_2_ (per kg body weight)			
		20 mg	40 mg	60 mg	75 mg
FRAP	19.0 ± 1.0	16.9 ± 0.9 (11)	14.7 ± 0.75* (22.6)	10.5 ± 0.53* (44.7)	8.7 ± 0.45* (54.2)
DPPH	75.6 ± 3.8	70.7 ± 3.5 (6.5)	63.1 ± 3.2* (16.5)	55.0 ± 2.8* (27.2)	51.7 ± 2.5* (31.6)

Fe(II)/mg protein and DPPH is in % quenching of DPPH radical. Values in parenthesis represent percent decrease from control.

Results are mean ± standard error of six different preparations. FRAP values are in μmoles

* Significantly different at p < 0.05 from control.

FRAP, ferric reducing antioxidant power; DPPH, 2,2-diphenyl-1-picrylhydrazyl.

### Enzymes of antioxidant defense

The activities of all major antioxidant defense enzymes decreased upon NaNO_2_ administration. The trend observed was dose-dependent with the extent of decline in the highest dose group being- SOD, 55%; CAT, 58.6%; TR, 57.1%; GR, 61.8% and GPx, 53.9% as compared to control group (Table 6).

**Table 6:** Effect of NaNO_2_ on the activities of major antioxidant defense enzymes in rat intestinal homogenates.

	Control	Dose of NaNO_2_ (per kg body weight)			
		20 mg	40 mg	60 mg	75 mg
SOD	41.6 ± 2.1	36.3 ± 1.8* (12.7)	31.2 ± 1.6* (25)	23.5 ± 1.3* (43.5)	18.7 ± 1.0* (55)
CAT	20.3 ± 1.0	17.7 ± 0.9* (12.8)	15.4 ± 0.8* (24.1)	11.3 ± 0.6* (44.3)	8.4 ± 0.4* (58.6)
TR	8.4 ± 0.43	7.8 ± 0.40 (7.1)	6.2 ± 0.30* (26.2)	4.2 ± 0.22* (50)	3.6 ± 0.20* (57.1)
GR	30.4 ± 1.7	26.0 ± 1.3* (14.5)	22.8 ± 1.1* (25)	15.7 ± 0.8* (48.4)	11.6 ± 0.6* (61.8)
GPx	20.4 ± 1.0	16.5 ± 0.9* (19.1)	13.5 ± 0.69* (33.8)	11.6 ± 0.60* (43.1)	9.4 ± 0.48* (53.9)

Specific activity of SOD is in units/mg protein/min, CAT is in μmoles/mg protein/min, TR, GR and GPx are in nmoles/mg protein/min. Values in parenthesis represent percent decrease from control.

Results are mean ± standard error of six different preparations.

* Significantly different at p < 0.05 from control.

SOD, Cu,Zn superoxide dismutase; CAT, catalase; TR, thioredoxin reductase; GR, glutathione reductase; GPx, glutathione reductase.

### Enzymes of carbohydrate metabolism

Enzymes of glycolysis, pentose phosphate pathway, gluconeogenesis and NADPH production were assayed in mucosal homogenates. Significant alterations in the activities of enzymes involved in carbohydrate metabolism and energy production were observed. The changes were again dose-dependent with maximum alterations evident in the highest dose group, HX, -45.1%; LDH- +152.6%; MDH, -55%; G6Pase, -35.7%; F6Pase, -44.1%; G6PD, -70.7% and ME, +187%, as compared to control group (Table 7).

**Table 7:** Effect of NaNO_2_ on the activities of carbohydrate metabolic enzymes in rat intestinal homogenates.

	Control	Dose of NaNO_2_ (per kg body weight)			
		20 mg	40 mg	60 mg	75 mg
HX	8.2 ± 0.43	7.8 ± 0.40 (-4.9)	6.1 ± 0.31* (-25.6)	5.0 ± 0.26* (-39)	4.5 ± 0.23* (-45.1)
LDH	19.6 ± 1.2	27.5 ± 1.3 (+40.3)	33.6 ± 1.8* (+71.4)	44.6 ± 2.3* (+127.6)	49.5 ± 2.5* (+152.6)
MDH	20.1 ± 1.0	16.7 ± 0.87* (-16.9)	11.8 ± 0.61* (-41.3)	10.1 ± 0.50* (-49.8)	9.0 ± 0.46* (-55)
G6Pase	4.2 ± 0.21	3.9 ± 0.20 (-7.1)	3.6 ± 0.18* (-14.3)	3.0 ± 0.16* (-28.6)	2.7 ± 0.13* (-35.7)
F6Pase	5.9 ± 0.3	5.6 ± 0.3 (-5.1)	4.9 ± 0.25* (-16.9)	3.6 ± 0.20* (-39)	3.3 ± 0.19* (-44.1)
G6PD	7.5 ± 0.40	6.4 ± 0.35* (-14.7)	5.1 ± 0.26* (-32)	2.8 ± 0.15* (-62.7)	2.2 ± 0.11* (-70.7)
ME	3.8 ± 0.20	4.3 ± 0.22* (+13.2)	6.3 ± 0.34* (+65.8)	8.8 ± 0.45* (+131.6)	10.9 ± 0.55* (+187)

Specific activities of HX, G6Pase and F6Pase are in μmoles/mg protein/h, LDH, MDH, G6PD and ME are in nmoles/mg protein/min. Values in parenthesis represent percent change from control.

Results are mean ± standard error of six different preparations.

* Significantly different at p < 0.05 from control.

HX, hexokinase; LDH, lactate dehydrogenase; MDH, malate dehydrogenase; G6Pase, glucose 6-phosphatase; F6Pase, fructose 1,6-bisphosphatase; G6PD, glucose 6-phosphate dehydrogenase; ME, malic enzyme.

### DNA damage

**Fig 3:**
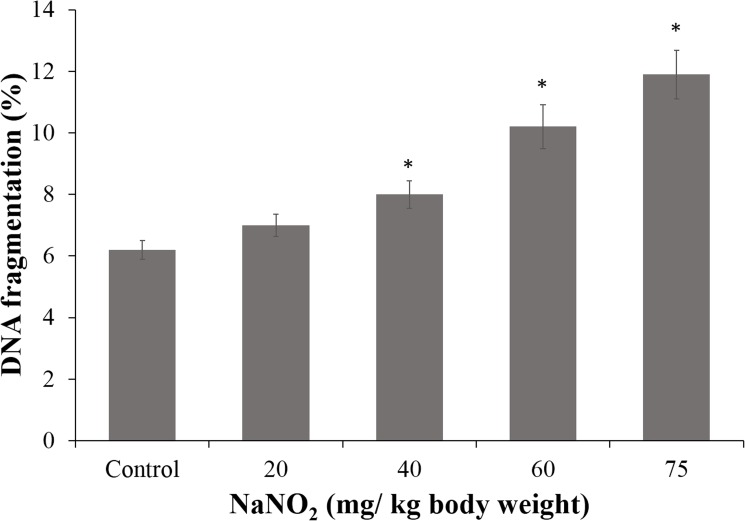
Diphenylamine assay. DNA fragmentation in intestinal mucosal cells of control and NaNO_2_-treated rats was determined. Results are mean ± standard error of six different samples. ^*^Significantly different at p< 0.05 from control.

NaNO_2_ administration increased DNA degradation and DNA-protein crosslinking in intestinal mucosal cells of animals in a dose dependent manner. DNA degradation was assessed by comet assay, agarose gel electrophoresis and colorimetric assay for release of free nucleotides. The diphenylamine assay showed that treatment with NaNO_2_ induced DNA damage in a dose-dependent manner in the intestinal cells of rats (Fig 3). DNA degradation was almost two times the control value in the group treated with the highest concentration of NaNO_2_ (75 mg/ kg body weight). This observation was further confirmed by agarose gel electrophoresis and the comet assay.

**Fig 4:**
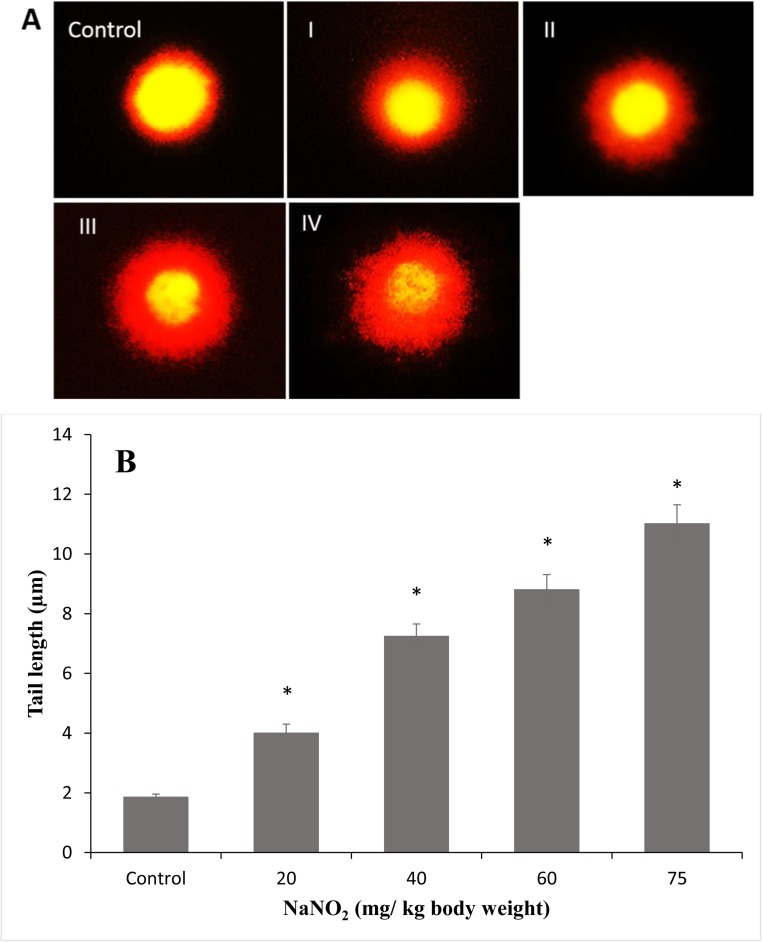
Comet assay. DNA damage in intestinal mucosal cells was studied by the comet assay as described in Materials and Methods. (A) DNA of cells were visualized under a fluorescent microscope and 25 comets scored per slide. Control; 20 mg/kg body weight NaNO_2_, I; 40 mg/kg body weight NaNO_2_, II; 60 mg/kg body weight NaNO_2_, III; 75 mg/kg body weight NaNO_2_, IV. (B) Comet tail lengths were recorded using the image analysis system, Komet 5.5, Kinetic Imaging, Liverpool, UK. Results are mean ± standard error of six different samples. ^*^Significantly different at p< 0.05 from control.

In the comet assay, a dose-responsive increase in tail length was observed in NaNO_2_ treated groups as compared to control group (Fig 4). This is indicative of DNA degradation and strand breaks. Migration length is considered to be directly related to fragment size and proportional to the level of single stranded breaks and alkali-labile sites [52].

**Fig 5:**
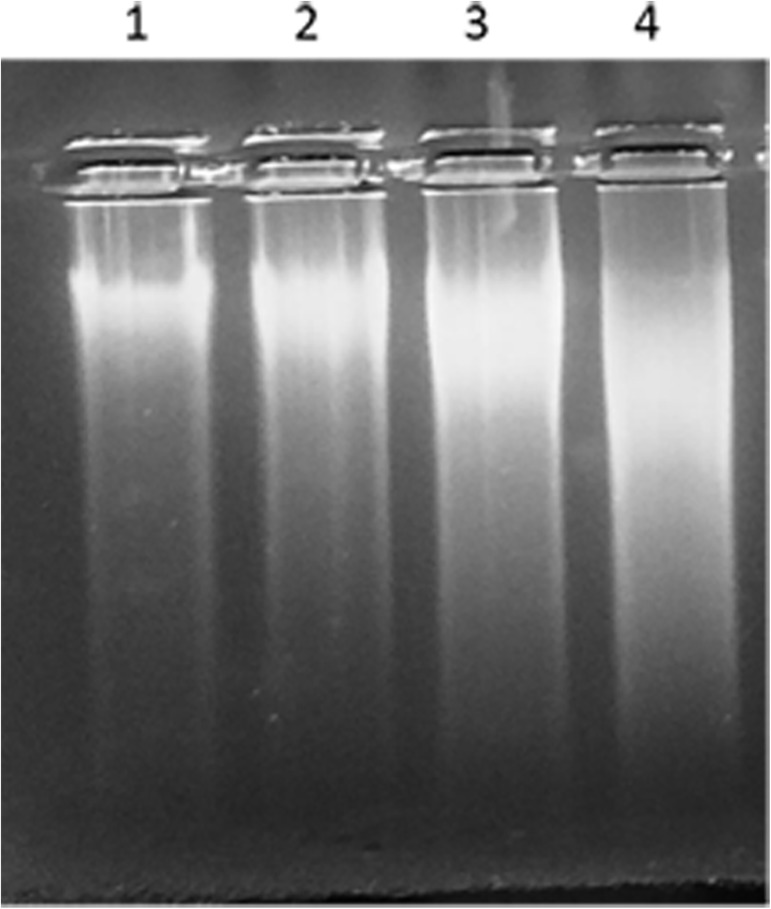
Agarose gel electrophoresis of DNA. The isolated DNA was electrophoresed on 0.7% agarose gels. Lane 1, control group; lane 2, 40 mg/kg body weight NaNO_2_; lane 3, 60 mg/kg body weight NaNO_2_; lane 4, 75 mg/kg body weight NaNO_2_.

Agarose gel electrophoresis of DNA from intestinal cells of rats showed significant smearing in a dose-dependent manner in NaNO_2_ treated groups as compared to control group (Fig 5). Smearing of DNA bands is a clear indication of DNA fragmentation and strand breaks. The highest dose group exhibited maximum smearing and hence, maximum damage. No evidence of 200 bp ladder formation, characteristic of apoptosis, was seen in any group.

An increase in DPCs induced by NaNO_2_ was clearly evident from the K^+^-SDS assay (Table 8). The percentage of DPC formation in the highest dose group was 3.4 times the value in the control group.

**Table 8:** Formation of DNA-protein crosslinks in intestine of rats treated with a single oral dose of NaNO_2_.

	DNA-protein crosslinks % ^a^	DNA-protein crosslinks coefficient ^b^
Control	2.8 ± 0.16	1.0
20 mg/kg body weight	3.36 ± 0.18	1.2
40 mg/kg body weight	5.82 ± 0.30^*^	2.1
60 mg/kg body weight	8.12 ± 0.42^*^	2.9
75 mg/kg body weight	9.41 ± 0.47^*^	3.36

^a^ DNA-protein crosslinks / total DNA

^b^ DNA-protein crosslinks (%) in NaNO_2_ treated animals / DNA-protein crosslinks (%) in control animals

Results are mean ± standard error of six different samples.

^*^ Significantly different at p< 0.05 from control.

### Histopathology

**Fig 6:**
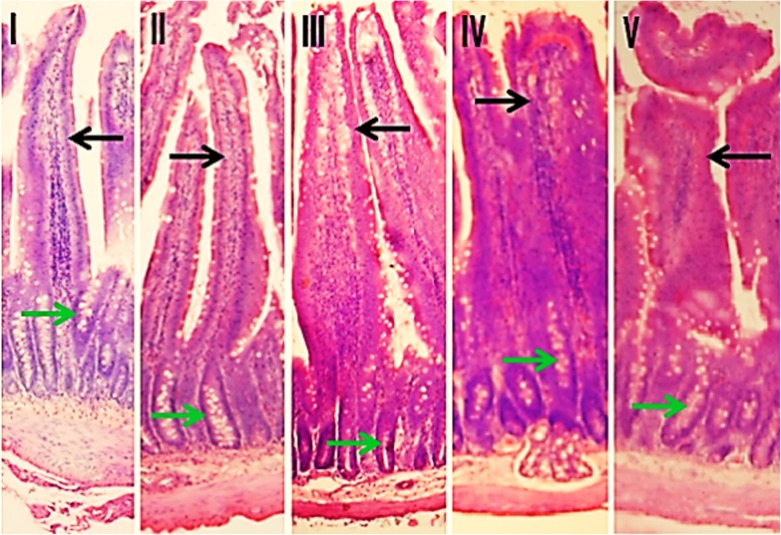
Histopathology of rat intestine. The image shows intestinal villi and crypt regions from duodenum of **[I]** control and NaNO_2_-administered groups **[II]** 20 mg **[III]** 40 mg **[IV]** 60 mg and **[V]** 75 mg/kg body weight. Black arrows indicate intestinal villi and green arrows indicate intestinal crypts. H and E stain, original magnification is 100x.

Histological examination of duodenal sections of rat intestine from control and treated groups revealed morphological alterations in NaNO_2_-treated groups (Fig 6). The changes were more prominent at higher dose and included swelling of villi, alteration in the contour, congestion, increased lymphocytic infiltration in the lamina propria with focal necrosis of enterocytes especially those located on the luminal half (apical) of villi. However, no obvious change in the crypt-to-villus ratio was observed.

## Discussion

Industrialization has contributed immensely to human development but at the same time has increased human exposure to many harmful pollutants and toxicants. NaNO_2_ is among one of these noxious contaminants which affects human and animal health across the globe. Rampant use of nitrogenous fertilizers to increase crop yield and improper waste disposal has mainly contributed to the nitrite load of the environment. Overexposure to nitrite has been reported to cause various health problems including cancer with contaminated water being the main source [53]. Nitrite acts as a pro-oxidant and pro-carcinogen at high doses. Nitrite intake through the oral route results in its rapid absorption through the gastrointestinal tract and entry into the bloodstream from where it is made available to other tissues. Once inside the cell, it is readily interconvertible to nitrate and/or NO through cellular oxidation/reduction processes [1]. Reactive oxygen species (ROS), generated by intracellular redox reactions, together with the formation of harmful compounds such as nitryl chloride, results in cytotoxicity and tissue damage [54]. In this work, we have investigated the effect of a single acute oral dose of NaNO_2_ on various biochemical aspects and morphology of rat intestine.

The BBM plays a key role in the digestive and absorptive functions of the intestine as it contains various digestive enzymes and transporters. A marked decrease (40–60%) in the activities of all BBM enzymes was evident in intestinal homogenates and isolated BBM vesicles from NaNO_2_ treated rats. The extent of decrease was almost the same in both preparations. This is clearly indicative of damage to the epithelial cell lining of the intestine. Kinetic studies performed show that the decline in enzyme activities was due to decrease in V_max_ with no significant alteration in K_M_ values for all the enzymes. Thus, nitrite administration did not affect the affinity of these enzymes for their substrates.

The decline in the activity of BBM enzymes can be attributed to a number of factors. Firstly, nitrite-generated ROS can cause oxidative modification of lipids and proteins which in turn will disrupt membrane integrity. This might cause loss of enzyme molecules from the epithelial cell lining. Secondly, ROS can directly oxidize enzyme molecules and decrease their activity. Higher protein oxidation was seen in mucosa of NaNO_2_-treated animals. Thirdly, nitrite anion itself can directly interact with enzymes resulting in their inhibition. This probability was inferred from the *in vitro* experiments performed with the enzymes.

The intestinal epithelial cells are constantly exposed to xenobiotics which makes them susceptible to oxidant attack and damage. As a defense, enterocytes are well-equipped with robust enzymatic and non-enzymatic antioxidant defenses which are important for their normal functioning [55]. NaNO_2_ administration led to a dose-dependent increase in levels of H_2_O_2_, a non-radical ROS. H_2_O_2_ can generate the vastly more damaging hydroxyl radical upon reaction with transition metals like iron. Increase in lipid peroxidation can be attributed to increase in the levels of superoxide radical and H_2_O_2_, since the activities of the enzymes that use them as substrates, SOD and CAT, were decreased. A two-fold increase in protein carbonyl content was observed in the highest NaNO_2_ dose group. ROS attack amino acids and form carbonyl groups; introduction of carbonyl groups is commonly used as an index of protein oxidation. NaNO_2_-induced protein oxidation and nitration of meat products has been reported earlier [56]. A profound decrease in levels of GSH and total SH content represents weakening of the first line of defense against oxidative damage of the cells. A reduction in GR activity, which reduces GSSG to GSH, could have resulted in lowered GSH levels. Direct oxidation of SH groups (of proteins and GSH) by ROS or formation of nitrosothiols upon reaction with NO could be another reason [57]. The thiol status of intestinal mucosa was, therefore, greatly affected upon NaNO_2_ administration.

The activities of all major antioxidant defense enzymes were found to dose-dependently decrease in all NaNO_2_-treated groups as compared to the control. ROS and free radicals are known to inactivate both SOD and CAT [58] which are metalloenzymes containing bound copper and iron, respectively. NO forms complexes with transition metal ions and can thus directly interact with these enzymes and interfere in their activity. Direct inhibition of CAT by nitrite in presence of specific anions has also been reported [59]. A decrease in the activities of TR, GR and GPx could also be due to oxidative modification of these enzymes. Peroxynitrite and superoxide radicals are specifically known to inhibit GPx activity [60].

A decrease in GSH content and inhibition of antioxidant enzymes can impair the antioxidant power of cells. A dose-dependent decrease in antioxidant capacity was observed in all NaNO_2_-treated groups. This strongly suggests oxidative damage by NaNO_2_ and inhibition of endogenous defense system of the cell. The lowered antioxidant capacity will make the cells more susceptible to oxidative damage by ROS, since the ROS quenching ability will be greatly compromised. Although antioxidant property of NaNO_2_ at low doses has been reported, it does not act as a direct antioxidant. Montenegro et al. [61] have shown the inability of nitrite to scavenge superoxide radicals or ameliorate iron-induced lipid peroxidation under *in vitro* conditions. The reported cellular antioxidant effect can probably be explained by NADPH oxidase downregulation and/or alterations in activities of enzymes involved in antioxidant defense [62,63].

Uptake of glucose from the diet and delivery to other tissues is mediated by the small intestine. The gut plays a key role in maintaining glucose homeostasis. The effect of NaNO_2_ administration on the activities of various enzymes involved in glucose utilization was examined. The activity of LDH was increased while MDH activity was decreased in all NaNO_2_ treated groups. LDH, an enzyme of anaerobic glycolysis, catalyzes the interconversion of pyruvate to lactate. Although lactate is a dead-end metabolite, necessitating reconversion to pyruvate, lactate production generates NAD^+^ which is required for glycolysis. MDH reversibly catalyzes malate oxidation to oxaloacetate which is an intermediate of the tricarboxylic acid (TCA) cycle and leads to energy production through the aerobic mode. Inhibition of MDH activity and increased LDH activity suggests a shift in energy production from aerobic to anaerobic mode of glycolysis. The first step in glucose utilization, whether by the aerobic, anaerobic or pentose-phosphate pathway, is catalyzed by HX. A decrease in HX activity in all NaNO_2_ treated groups indicates decreased cellular energy production. The role of intestine in gluconeogenesis has been recently discovered. Although liver is the main site of gluconeogenesis in normal state, intestinal gluconeogenesis is important during starvation and anhepatic conditions [64]. G6Pase is an endoplasmic reticulum-associated enzyme which catalyzes the final step of gluconeogenesis and glycogenolysis. F6Pase is involved in gluconeogenesis and the Calvin cycle both of which are anabolic pathways. Intestinal G6Pase and F6Pase, therefore, play key role in the homeostatic regulation of blood glucose levels and NaNO_2_ decreased the activities of both enzymes. Membrane disruption and oxidative modification could be possible reasons. Lowered G6PD activity and increased ME activity was found in NaNO_2_ treated groups. G6PD is the first and an indispensable enzyme of the pentose phosphate pathway. It generates NADPH which is the major cellular reducing equivalent. A decrease in G6PD activity makes the cell more susceptible to oxidant attack. Increased activity of ME, which also generates reducing equivalents, can be considered as an adaptive response to compensate the decrease in G6PD activity.

Increased carbonyl content of proteins can lead to their enhanced crosslinking with DNA. A DPC is created when a protein becomes covalently bound to DNA. These lesions interfere in DNA replication, transcription and repair which might lead to permanent DNA damage [65]. A dose-dependent increase in DPC formation in all NaNO_2_-treated groups was observed. DPCs greatly interfere in DNA-repair mechanisms resulting in strand breaks, fragmentation and other genetic lesions. NaNO_2_-mediated DNA damage could be due to several mechanisms: direct chemical modification by free radicals [66] or indirectly through free radical induced lipid oxidation products like unsaturated aldehydes and malondialdehyde that can bind to DNA to generate mutagenic lesions [67]. Our results comply with previous reports which showed that nitrite and its related metabolized products cause DNA damage and genotoxicity [68,69]. Nitrite can cross membranes, probably as nitrous acid, a known mutagenic compound that can damage DNA through several mechanisms [70,71]. Nitrite itself has been shown to damage DNA under *in vitro* conditions in human respiratory tract cell lines and calf-thymus DNA [72,73]. NO also causes dose-responsive DNA damage [74,75], while peroxynitrite and other higher oxides of nitrogen are also known to readily attack and damage DNA [76]. Several reactive nitrogen species have been implicated in the multistage carcinogenesis process associated with chronic inflammation and infections [77,78].

Lowered ATPase activity in NaNO_2_ treated animals suggests that the basolateral membrane of intestinal cells was damaged. The establishment of an electrochemical gradient of sodium ions across the epithelial cell boundary of the lumen is probably the most important process that aids absorption. To maintain low intracellular levels of sodium, a large number of Na,K- ATPases, highly conserved integral membrane proteins, are present in the basolateral membrane of enterocytes [79]. ATPases maintain a gradient of both sodium concentration and charge facilitating absorption of sugar, oligopeptide and amino acids [80]. A decline in the activity of this enzyme would lead to derangement of the primary function of absorption of enterocytes which would impair overall balance of solutes and ions in the body. ACP is a lysosomal marker enzyme and an increase in its activity is indicative of intestinal damage. These changes may be attributed to free radical attack or nitrosation of essential sulfhydryl groups (ATPase) by NO. Lipid and protein oxidation might have also contributed to membrane damage and consequent enzyme inhibition.

The nitrite-induced intestinal damage suggested by the biochemical changes observed above was confirmed by histological studies. Morphological changes in the intestinal epithelial cells were studied by microscopic examination of stained sections of duodenum of control and NaNO_2_ treated groups. In the higher dose groups, marked changes were obvious, including lymphocytic infiltration and congestion in the lamina propria and focal necrosis of enterocytes located in the apical region of villi (Fig 6).

**Fig 7:**
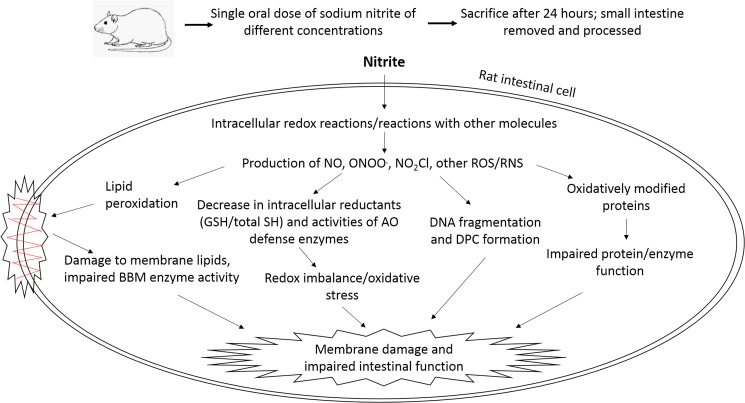
Schematic representation of NaNO_2_-induced intestinal damage. AO, antioxidant; DPC, DNA-protein crosslinks; GSH, reduced glutathione; BBM, brush border membrane; NO, nitric oxide; ONOO^.^, peroxynitrite; NO_2_Cl, nitryl chloride; ROS/RNS, reactive oxygen/nitrogen species.

Thus, nitrite administration caused marked changes in various biochemical parameters and morphology of rat intestinal cells. The NaNO_2_-induced damage to intestinal epithelial cells can affect overall physiology in several ways. 1. Decreased activities of BBM enzymes and total ATPase will reduce absorption of nutrients, especially sugars and amino acids, into the epithelial cells of the small intestine. This will reduce the pool of essential building blocks of the body which will, in turn, greatly hamper energy production. 2. Alterations in the activities of enzymes of carbohydrate metabolism will affect glucose utilization by enterocytes and impair intestinal gluconeogenesis which plays key role in energy homeostasis [81]. 3. Decreased antioxidant capacity of enterocytes due to impairment of its enzymatic and non-enzymatic components will increase susceptibility to oxidant attack. 4) DNA lesions of various nature may cause genotoxic and/or mutagenic effects which may lead to cancer development. Oxidative stress condition is considered to be a major etiologic factor in several intestinal diseases [82]. A cumulative effect of all these factors might have led to intestinal damage in NaNO_2_-treated rats as observed in our study. A schematic representation of the damaging effects of NaNO_2_ on rat intestine, based on our experimental observations, is shown in Fig 7.

An over view of the damaging effects of NaNO_2_ on intestinal cells, as presented in this study, will be helpful in deciphering the mechanism of nitrite toxicity in greater detail, understanding the role that nitrite plays in cancer development and other pathologies and in the choice of protective/ therapeutic agents to counter its deleterious effects.

## References

[1] Lundberg JO, Weitzberg E, Gladwin MT. The nitrate-nitrite-nitric oxide pathway in physiology and therapeutics. Nat Rev Drug Discov. 2008;7: 156–167. doi: 10.1038/nrd2466 18167491 10.1038/nrd2466

[2] Hord NG, Tang Y, Bryan NS. Food sources of nitrates and nitrites: the physiologic context for potential health benefits. Am J Clin Nutr. 2009;90: 1–10. doi: 10.3945/ajcn.2008.27131 19439460 10.3945/ajcn.2008.27131

[3] Hord NG. Dietary nitrates, nitrites, and cardiovascular disease. Curr Atheroscler Rep. 2011;13: 484–492. doi: 10.1007/s11883-011-0209-9 21968645 10.1007/s11883-011-0209-9

[4] McNally B, Griffin JL, Roberts LD. Dietary inorganic nitrate: From villain to hero in metabolic disease? Mol Nutr Food Res. 2016;60: 67–78. doi: 10.1002/mnfr.201500153 26227946 10.1002/mnfr.201500153 PMC4863140

[5] Galloway JN, Aber JD, Erisman JW, Seitzinger SP, Howarth RW, Cowling EB, et al The nitrogen cascade. BioScience. 2003;53: 341–356.

[6] WHO. Nitrate and nitrite in drinking-water: WHO guidelines for drinking-water quality. 2011; 1–31.

[7] Lawniczak AE, Zbierska J, Nowak B, Achtenberg K, Grześkowiak A, Kanas K. Impact of agriculture and land use on nitrate contamination in groundwater and running waters in central-west Poland. Environ Monit Assess. 2016;188. 26887311 10.1007/s10661-016-5167-9 PMC4757607

[8] Kaplan A, Smith C, Promnitz DA, Joffe BI, Seftel HC. Methaemoglobinaemia due to accidental sodium nitrite poisoning. Report of 10 cases. S Afr Med J. 1990;77: 300–301. 2315812

[9] Chui JSW, Poon WT, Chan KC, Chan AYW, Buckley TA. Nitrite-induced methaemoglobinaemia—aetiology, diagnosis and treatment. Anaesthesia. 2005;60: 496–500. doi: 10.1111/j.1365-2044.2004.04076.x 15819771 10.1111/j.1365-2044.2004.04076.x

[10] Bryan NS, Loscalzo J, editors. Nitrite and nitrate in human health and disease [Internet]. Totowa, NJ: Humana Press; 2011 Available: http://link.springer.com/10.1007/978-1-60761-616-0

[11] Gui G, Meng S, Li L, Liu B, Liang H, Huangfu C. Sodium nitrite enhanced the potentials of migration and invasion of human hepatocellular carcinoma SMMC-7721 cells through induction of mitophagy. Yao Xue Xue Bao. 2016;51: 59–67. 27405163

[12] Zhou L, Zahid M, Anwar MM, Pennington KL, Cohen SM, Wisecarver JL, et al Suggestive evidence for the induction of colonic aberrant crypts in mice fed sodium nitrite. Nutr Cancer. 2016;68: 105–112. doi: 10.1080/01635581.2016.1102298 26699517 10.1080/01635581.2016.1102298

[13] Lundberg JO, Weitzberg E, Cole JA, Benjamin N. Nitrate, bacteria and human health. Nat Rev Microbiol. 2004;2: 593–602. doi: 10.1038/nrmicro929 15197394 10.1038/nrmicro929

[14] Sen NP, Seaman SW, Baddoo PA, Burgess C, Weber D. Formation of N-nitroso-N-methylurea in various samples of smoked/dried fish, fish sauce, seafoods, and ethnic fermented/pickled vegetables following incubation with nitrite under acidic conditions. J Agric Food Chem. 2001;49: 2096–2103. 11308373 10.1021/jf0011384

[15] Ohshima H, Yoshie Y, Auriol S, Gilibert I. Antioxidant and pro-oxidant actions of flavonoids: effects on DNA damage induced by nitric oxide, peroxynitrite and nitroxyl anion. Free Radic Biol Med. 1998;25: 1057–1065. 9870559 10.1016/s0891-5849(98)00141-5

[16] NTP. Toxicology and carcinogenesis studies of sodium nitrite (CAS NO. 7632-00-0) in F344/N rats and B6C3F1 mice (drinking water studies). Natl Toxicol Program Tech Rep Ser. 2001;495: 7–273. 12563346

[17] Ansari FA, Mahmood R. Sodium nitrite enhances generation of reactive oxygen species that decrease antioxidant power and inhibit plasma membrane redox system of human erythrocytes. Cell Biol Int. 2016;40: 887–894. doi: 10.1002/cbin.10628 27214747 10.1002/cbin.10628

[18] Jia R, Han C, Lei JL, Liu BL, Huang B, Huo HH, et al Effects of nitrite exposure on haematological parameters, oxidative stress and apoptosis in juvenile turbot (Scophthalmus maximus). Aquat Toxicol Amst Neth. 2015;169: 1–9. 10.1016/j.aquatox.2015.09.016 26476021

[19] Al-Gayyar MMH, Hassan HM, Alyoussef A, Abbas A, Darweish MM, El-Hawwary AA. Nigella sativa oil attenuates chronic nephrotoxicity induced by oral sodium nitrite: Effects on tissue fibrosis and apoptosis. Redox Rep Commun Free Radic Res. 2016;21: 50–60. 10.1179/1351000215Y.0000000035 PMC6837667 26221999

[20] Sherif IO, Al-Gayyar MMH. Antioxidant, anti-inflammatory and hepatoprotective effects of silymarin on hepatic dysfunction induced by sodium nitrite. Eur Cytokine Netw. 2013;24: 114–121. doi: 10.1684/ecn.2013.0341 24225033 10.1684/ecn.2013.0341

[21] Özen H, Kamber U, Karaman M, Gül S, Atakişi E, Özcan K, et al Histopathologic, biochemical and genotoxic investigations on chronic sodium nitrite toxicity in mice. Exp Toxicol Pathol. 2014;66: 367–375. doi: 10.1016/j.etp.2014.05.003 24947405 10.1016/j.etp.2014.05.003

[22] Katabami K, Hayakawa M, Gando S. Severe methemoglobinemia due to sodium nitrite poisoning. Case Rep Emerg Med. 2016;2016: e9013816. 10.1155/2016/9013816 PMC4987464 27563472

[23] Sohn CH, Seo DW, Ryoo SM, Lee JH, Kim WY, Lim KS, et al Life-threatening methemoglobinemia after unintentional ingestion of antifreeze admixtures containing sodium nitrite in the construction sites. Clin Toxicol Phila Pa. 2014;52: 44–47. 10.3109/15563650.2013.863327 24266433

[24] Farooq N, Yusufi ANK, Mahmood R. Effect of fasting on enzymes of carbohydrate metabolism and brush border membrane in rat intestine. Nutr Res. 2004;24: 407–416.

[25] Lowry OH, Rosebrough NJ, Farr AL, Randall RJ. Protein measurement with the Folin phenol reagent. J Biol Chem. 1951;193: 265–275. 14907713

[26] Slaoui M, Fiette L. Histopathology procedures: from tissue sampling to histopathological evaluation. Methods Mol Biol. 2011;691: 69–82. doi: 10.1007/978-1-60761-849-2_4 20972747 10.1007/978-1-60761-849-2_4

[27] Glossmann H, Neville DM. γ-Glutamyltransferase in kidney brush border membranes. FEBS Lett. 1972;19: 340–344. 11946246 10.1016/0014-5793(72)80075-9

[28] Goldmann DR, Schlesinger H, Segal S. Isolation and characterization of the brush border fraction from newborn rat renal proximal tubule cells. Biochim Biophys Acta BBA-Biomembr. 1976;419: 251–260. 10.1016/0005-2736(76)90351-5 2293

[29] Kempson SA, Kim JK, Northrup TE, Knox FG, Dousa TP. Alkaline phosphatase in adaptation to low dietary phosphate intake. Am J Physiol-Gastrointest Liver Physiol. 1979;237: G465–G473. 10.1152/ajpendo.1979.237.5.E465 495749

[30] Miller GL. Use of dinitrosalicylic acid reagent for determination of reducing sugar. Anal Chem. 1959;31: 426–428.

[31] Buege JA, Aust SD. Microsomal lipid peroxidation. Methods Enzymol. 1978;52: 302–310. 672633 10.1016/s0076-6879(78)52032-6

[32] Levine RL, Garland D, Oliver CN, Amici A, Climent I, Lenz AG, et al Determination of carbonyl content in oxidatively modified proteins. Methods Enzymol. 1990;186: 464–478. 1978225 10.1016/0076-6879(90)86141-h

[33] Hissin PJ, Hilf R. A fluorometric method for determination of oxidized and reduced glutathione in tissues. Anal Biochem. 1976;74: 214–226. 962076 10.1016/0003-2697(76)90326-2

[34] Sedlak J, Lindsay RH. Estimation of total, protein-bound, and nonprotein sulfhydryl groups in tissue with Ellman’s reagent. Anal Biochem. 1968;25: 192–205. 4973948 10.1016/0003-2697(68)90092-4

[35] Gay C, Gebicki JM. A critical evaluation of the effect of sorbitol on the ferric-xylenol orange hydroperoxide assay. Anal Biochem. 2000;284: 217–220. doi: 10.1006/abio.2000.4696 10964403 10.1006/abio.2000.4696

[36] Mohrenweiser HW, Novotny JE. ACP1GUA-1—a low-activity variant of human erythrocyte acid phosphatase: association with increased glutathione reductase activity. Am J Hum Genet. 1982;34: 425–433. 7081221 PMC1685341

[37] Bonting SL, Simon KA, Hawkins NM. Studies on sodium-potassium-activated adenosine triphosphatase: I. Quantitative distribution in several tissues of the cat. Arch Biochem Biophys. 1961;95: 416–423. 13871109 10.1016/0003-9861(61)90170-9

[38] Benzie IFF, Strain JJ. The ferric reducing ability of plasma (FRAP) as a measure of “antioxidant power”: The FRAP assay. Anal Biochem. 1996;239: 70–76. doi: 10.1006/abio.1996.0292 8660627 10.1006/abio.1996.0292

[39] Mishra K, Ojha H, Chaudhury NK. Estimation of antiradical properties of antioxidants using DPPH assay: A critical review and results. Food Chem. 2012;130: 1036–1043.

[40] Marklund S, Marklund G. Involvement of the superoxide anion radical in the autoxidation of pyrogallol and a convenient assay for superoxide dismutase. Eur J Biochem. 1974;47: 469–474. 4215654 10.1111/j.1432-1033.1974.tb03714.x

[41] Aebi H. Catalase in vitro. Methods Enzymol. 1984;105: 121–126. 6727660 10.1016/s0076-6879(84)05016-3

[42] Mannervik B, Carlberg I. Glutathione reductase. Methods Enzymol. 1985;113: 484–490. 3003504 10.1016/s0076-6879(85)13062-4

[43] Tamura T, Stadtman TC. A new selenoprotein from human lung adenocarcinoma cells: purification, properties, and thioredoxin reductase activity. Proc Natl Acad Sci U S A. 1996;93: 1006–1011. 8577704 10.1073/pnas.93.3.1006 PMC40020

[44] Flohé L, Günzler WA. Assays of glutathione peroxidase. Methods Enzymol. 1984;105: 114–120. 6727659 10.1016/s0076-6879(84)05015-1

[45] Khundmiri SJ, Asghar M, Khan F, Salim S, Yusufi AN. Effect of ischemia and reperfusion on enzymes of carbohydrate metabolism in rat kidney. J Nephrol. 2004;17: 377–383. 15365957

[46] Crane RK, Sols A. The association of hexokinase with particulate fractions of brain and other tissue homogenates. J Biol Chem. 1953;203: 273–292. 13069512

[47] Shonk CE, Boxer GE. Enzyme patterns in human tissues. I. Methods for the determination of glycolytic enzymes. Cancer Res. 1964;24: 709–721. 14188477

[48] Singh NP, McCoy MT, Tice RR, Schneider EL. A simple technique for quantitation of low levels of DNA damage in individual cells. Exp Cell Res. 1988;175: 184–191. 3345800 10.1016/0014-4827(88)90265-0

[49] Burton K. A study of the conditions and mechanism of the diphenylamine reaction for the colorimetric estimation of deoxyribonucleic acid. Biochem J. 1956;62: 315–323. 13293190 10.1042/bj0620315 PMC1215910

[50] Evans GA. Molecular cloning: A laboratory manual. Second edition. Volumes 1, 2, and 3. Current protocols in molecular biology. Volumes 1 and 2. Cell. 1990;61: 17–18.

[51] Zhitkovich A, Costa M. A simple, sensitive assay to detect DNA-protein crosslinks in intact cells and in vivo. Carcinogenesis. 1992;13: 1485–1489. 1499101 10.1093/carcin/13.8.1485

[52] Tice RR, Agurell E, Anderson D, Burlinson B, Hartmann A, Kobayashi H, et al Single cell gel/comet assay: guidelines for in vitro and in vivo genetic toxicology testing. Environ Mol Mutagen. 2000;35: 206–221. 10737956 10.1002/(sici)1098-2280(2000)35:3<206::aid-em8>3.0.co;2-j

[53] Archer MC. Hazards of nitrate, nitrite and N-nitroso compounds in human nutrition. Nutr Toxicol. 2012;1: 327.

[54] Eiserich JP, Cross CE, Jones AD, Halliwell B, Vliet A van der. Formation of nitrating and chlorinating species by reaction of nitrite with hypochlorous Acid A NOVEL MECHANISM FOR NITRIC OXIDE-MEDIATED PROTEIN MODIFICATION. J Biol Chem. 1996;271: 19199–19208. 8702599 10.1074/jbc.271.32.19199

[55] Circu ML, Aw TY. Redox biology of the intestine. Free Radic Res. 2011;45: 1245–1266. doi: 10.3109/10715762.2011.611509 21831010 10.3109/10715762.2011.611509 PMC3210416

[56] Vossen E, De Smet S. Protein oxidation and protein nitration influenced by sodium nitrite in two different meat model systems. J Agric Food Chem. 2015;63: 2550–2556. doi: 10.1021/jf505775u 25700017 10.1021/jf505775u

[57] Radi R. Nitric oxide, oxidants, and protein tyrosine nitration. Proc Natl Acad Sci U S A. 2004;101: 4003–4008. doi: 10.1073/pnas.0307446101 15020765 10.1073/pnas.0307446101 PMC384685

[58] Escobar JA, Rubio MA, Lissi EA. SOD and catalase inactivation by singlet oxygen and peroxyl radicals. Free Radic Biol Med. 1996;20: 285–290. 8720898 10.1016/0891-5849(95)02037-3

[59] Titov VY, Petrenko YM. Nitrite-catalase interaction as an important element of nitrite toxicity. Biochemistry (Mosc). 2003;68: 627–633. 12943506 10.1023/a:1024609624652

[60] Pigeolet E, Corbisier P, Houbion A, Lambert D, Michiels C, Raes M, et al Glutathione peroxidase, superoxide dismutase, and catalase inactivation by peroxides and oxygen derived free radicals. Mech Ageing Dev. 1990;51: 283–297. 2308398 10.1016/0047-6374(90)90078-t

[61] Montenegro MF, Pinheiro LC, Amaral JH, Marçal DMO, Palei ACT, Costa-Filho AJ, et al Antihypertensive and antioxidant effects of a single daily dose of sodium nitrite in a model of renovascular hypertension. Naunyn Schmiedebergs Arch Pharmacol. 2012;385: 509–517. doi: 10.1007/s00210-011-0712-0 22262021 10.1007/s00210-011-0712-0

[62] Singh M, Arya A, Kumar R, Bhargava K, Sethy NK. Dietary nitrite attenuates oxidative stress and activates antioxidant genes in rat heart during hypobaric hypoxia. Nitric Oxide Biol Chem. 2012;26: 61–73. 10.1016/j.niox.2011.12.002 22197744

[63] Montenegro MF, Amaral JH, Pinheiro LC, Sakamoto EK, Ferreira GC, Reis RI, et al Sodium nitrite downregulates vascular NADPH oxidase and exerts antihypertensive effects in hypertension. Free Radic Biol Med. 2011;51: 144–152. doi: 10.1016/j.freeradbiomed.2011.04.005 21530643 10.1016/j.freeradbiomed.2011.04.005

[64] Penhoat A, Fayard L, Stefanutti A, Mithieux G, Rajas F. Intestinal gluconeogenesis is crucial to maintain a physiological fasting glycemia in the absence of hepatic glucose production in mice. Metabolism. 2014;63: 104–111. doi: 10.1016/j.metabol.2013.09.005 24135501 10.1016/j.metabol.2013.09.005

[65] Barker S, Weinfeld M, Murray D. DNA-protein crosslinks: their induction, repair, and biological consequences. Mutat Res. 2005;589: 111–135. doi: 10.1016/j.mrrev.2004.11.003 15795165 10.1016/j.mrrev.2004.11.003

[66] Halliwell B, Aruoma OI. DNA damage by oxygen-derived species. Its mechanism and measurement in mammalian systems. FEBS Lett. 1991;281: 9–19. 1849843 10.1016/0014-5793(91)80347-6

[67] Marnett LJ. Oxy radicals, lipid peroxidation and DNA damage. Toxicology. 2002;181–182: 219–222. 12505314 10.1016/s0300-483x(02)00448-1

[68] Luca D, Luca V, Cotor F, Răileanu L. In vivo and in vitro cytogenetic damage induced by sodium nitrite. Mutat Res. 1987;189: 333–339. 3670336 10.1016/0165-1218(87)90065-6

[69] Rubenchik BL, Osinkovskaya ND, Mikhailenko VM, Furman MA, Boim TM (Laboratory of E and C. The carcinogenic danger of nitrite pollution of the environment. J Environ Pathol Toxicol Oncol U S. 1990;10:6 Available: http://www.osti.gov/scitech/biblio/5590685 2095413

[70] Shingles R, Roh MH, McCarty RE. Direct measurement of nitrite transport across erythrocyte membrane vesicles using the fluorescent probe, 6-methoxy-N-(3-sulfopropyl) quinolinium. J Bioenerg Biomembr. 1997;29: 611–616. 9559862 10.1023/a:1022491220299

[71] Hartman Z, Henrikson EN, Hartman PE, Cebula TA. Molecular models that may account for nitrous acid mutagenesis in organisms containing double-stranded DNA. Environ Mol Mutagen. 1994;24: 168–175. 7957120 10.1002/em.2850240305

[72] Spencer JP, Whiteman M, Jenner A, Halliwell B. Nitrite-induced deamination and hypochlorite-induced oxidation of DNA in intact human respiratory tract epithelial cells. Free Radic Biol Med. 2000;28: 1039–1050. 10832065 10.1016/s0891-5849(00)00190-8

[73] Zhao K, Whiteman M, Spencer JP, Halliwell B. DNA damage by nitrite and peroxynitrite: protection by dietary phenols. Methods Enzymol. 2001;335: 296–307. 11400378 10.1016/s0076-6879(01)35252-7

[74] Nguyen T, Brunson D, Crespi CL, Penman BW, Wishnok JS, Tannenbaum SR. DNA damage and mutation in human cells exposed to nitric oxide in vitro. Proc Natl Acad Sci U S A. 1992;89: 3030–3034. 1557408 10.1073/pnas.89.7.3030 PMC48797

[75] Tamir S, Burney S, Tannenbaum SR. DNA damage by nitric oxide. Chem Res Toxicol. 1996;9: 821–827. doi: 10.1021/tx9600311 8828916 10.1021/tx9600311

[76] Wiseman H, Halliwell B. Damage to DNA by reactive oxygen and nitrogen species: role in inflammatory disease and progression to cancer. Biochem J. 1996;313 (Pt 1): 17–29. 8546679 10.1042/bj3130017 PMC1216878

[77] Payne CM, Bernstein C, Bernstein H, Gerner EW, Garewal H. Reactive nitrogen species in colon carcinogenesis. Antioxid Redox Signal. 1999;1: 449–467. doi: 10.1089/ars.1999.1.4-449 11233144 10.1089/ars.1999.1.4-449

[78] Sawa T, Ohshima H. Nitrative DNA damage in inflammation and its possible role in carcinogenesis. Nitric Oxide Biol Chem Off J Nitric Oxide Soc. 2006;14: 91–100. 10.1016/j.niox.2005.06.005 16099698

[79] Quigley JP, Gotterer GS. Distribution of (Na+-K+-stimulated ATPase activity in rat intestinal mucosa. Biochim Biophys Acta BBA—Biomembr. 1969;173: 456–468. 10.1016/0005-2736(69)90010-8 4305976

[80] Barrett KE, Ghishan FK, Merchant JL, Said HM, Wood JD. Physiology of the Gastrointestinal Tract. Academic Press; 2006.

[81] Mithieux G, Gautier-Stein A. Intestinal glucose metabolism revisited. Diabetes Res Clin Pract. 2014;105: 295–301. doi: 10.1016/j.diabres.2014.04.008 24969963 10.1016/j.diabres.2014.04.008

[82] Moura FA, de Andrade KQ, dos Santos JCF, Araújo ORP, Goulart MOF. Antioxidant therapy for treatment of inflammatory bowel disease: Does it work? Redox Biol. 2015;6: 617–639. doi: 10.1016/j.redox.2015.10.006 26520808 10.1016/j.redox.2015.10.006 PMC4637335

